# Family functioning in the aftermath of a natural disaster

**DOI:** 10.1186/1471-244X-12-55

**Published:** 2012-07-10

**Authors:** Brett M McDermott, Vanessa E Cobham

**Affiliations:** 1 Kids in Mind Research: The Mater Center for Service Research in Mental Health, Brisbane, Queensland, Australia; 2 Mater Medical Research Institute, Brisbane, Queensland, Australia; 3 Department of Psychology, University of Queensland, Brisbane, Queensland, Australia

## Abstract

**Background:**

Increased understanding of the complex determinants of adverse child mental health outcomes following acute stress such as natural disasters has led to a resurgence of interest in the role of parent psychopathology and parenting. The authors investigated whether family functioning in the post-disaster environment would be impaired relative to a non-exposed sample and potential correlates with family functioning such as disaster-related exposure and child posttraumatic mental health symptoms.

**Methods:**

Three months after a category 5 tropical cyclone that impacted north Queensland Australia, school-based screening was undertaken to case identify children who may benefit from a mental health intervention. Along with obtaining informed consent, parents completed a measure of family functioning.

**Results:**

Of 145 families of children aged 8 to 12 years, 28.3% met criteria for dysfunction on the Family Adjustment Device, double the frequency in a community sample. The dysfunction group was significantly more likely to have experienced more internalising (anxiety/depression) symptoms. However, in an adjusted logistic regression model this group were not more likely to have elevated disaster-related exposure nor did children in these families validate more PTSD symptoms.

**Conclusions:**

The implications of post-disaster discordant family functioning and possible different causal pathways for depressive and PTSD-related symptomatic responses to traumatic events are discussed.

## Background

In a review of disaster literature since 1980 Galea, Nandi, and Vlahov [1] reported the estimated prevalence of post-traumatic stress disorder (PTSD) in adults following natural disasters varied between 5 and 60 percent within the first two years post disaster. There is also clear evidence that children and adolescents are vulnerable [1]. La Greca and Prinstein in a review of natural disaster studies reported that 30-50% of effected children and adolescents demonstrate moderate to severe symptoms of PTSD, while 5-10% meet criteria for a full PTSD diagnosis [2]. PTSD has tended to be the primary psychological outcome assessed in children and adolescents following a natural disaster, with depression and other anxiety disorders sometimes assessed. For example, three months after an earthquake, a school based study found PTSD was present in 4.3% of the sample, while clinical depression was present in 13.9% [3]. Research after man-made, often terrorism-related disasters has emphasised the range of possible short and long term emotional outcomes [4,5]. A unique study in which pre-disaster mental health ratings were able to be compared with post-disaster ratings highlights the need to broaden the scope to include factors such as aggression and alcohol use [6]. It has also been suggested that natural disasters represent a particular challenge to youth [7]. Children and adolescents are less likely than adults to have the cognitive and emotional maturity to effectively respond to post-disaster challenges and thus must often rely for support on the significant adults in their lives. Because these adults will typically have been affected by the same disaster, their capacity to support children and adolescents is often significantly reduced. The functioning of a child or adolescent’s family unit following a natural disaster would thus seem to be of critical importance.

Recently, there has been a resurgence of interest in the role of parent psychopathology and parenting variables in child PTSD post-disaster. Indeed Spell and colleagues reported correlations between maternal psychological distress and posttraumatic stress, and PTSD and internalising symptoms in children exposed to Hurricane Katrina [8].However, post-disaster family functioning per se has remained a relatively neglected area. Hobfoll’s Conservation of Resources Theory [9] provides a useful framework for thinking about the importance of post-disaster family functioning. According to this theory, psychological stress occurs when there is a threat of resource loss (where resources may be either material or psychological), an actual resource loss, or a lack of resource gain following a significant investment of resources. For people exposed to a natural disaster there is typically a chain of losses, with the first link being the traumatic events associated with the disaster itself. Next are negative events that occur following the disaster – such as loss of one’s home and one’s livelihood – that intensify the crisis. These added stressors are hypothesised to result in a general deterioration in family relationships and functioning, as individuals’ capacity to cope becomes stretched. For children and adolescents, family-related resources are hypothesised to be the most important resources [10]. Parents in post-disaster environments may reach a point where they are not able to provide their children with sufficient attention, support, and care [11]. The research constructs related to parents and parenting in past disaster settings include parental support [10,12,13]; parental over-protectiveness [10]; parental psychopathology [8,14-17]; and reduced parenting efficacy [18] as predictors of post-disaster child PTSD symptoms. McDermott and colleagues investigated family resilience; the ability of the family to respond positively to an adverse situation, and reported low family resilience was related to post-disaster child anxiety and depressive symptoms but not to child PTSD [19].

Clearly, there is considerable overlap between parenting style, parenting efficacy and family functioning. However, family functioning tends to be conceptualized more broadly, typically including the domains contained in the McMaster Family Assessment Model – behaviour control; communication, affective responsiveness, problem solving, roles and affective involvement. To our knowledge, very few studies have assessed post-disaster family functioning. McFarlane reported that eight months after a bushfire, exposed and non-exposed families were distinguished by increased levels of irritability, withdrawal, and conflict between family members [16]. In this study disruptions to family functioning were a greater predictor of child post-traumatic phenomena than were parent-report of exposure or loss of possessions. Limitations include ‘disruption to family functioning’ was not assessed by a psychometrically sound and purpose designed measure of family functioning. Further, parent-report of child PTSD psychopathology has well established limitations. Green and colleagues looked at predictors of “probable” PTSD in 179 children aged between 2 and 15 years exposed to the Buffalo Dam creek collapse [14]. This research is characterized by intrinsic methodological challenges including extrapolation of information from lawsuit evaluation reports to make PTSD diagnoses rather than any direct measure of child PTSD. Green et al. reported that an “irritable” family atmosphere predicted PTSD symptoms in children over and above individual parents’ psychological functioning [14]. More recently, Kilic and colleagues assessed 35 families living in a ‘tent city’ following the 1999 earthquake in Turkey for mental health symptoms - specifically, PTSD, depression and state and trait anxiety [15]. These researchers reported that PTSD in children was predicted by female gender, paternal PTSD and paternal depression scores. Depression in children on the other hand was predicted by paternal PTSD status only. State and trait anxiety in children was predicted by lower family functioning, which was not related to either child PTSD or Depression. Kilic et al., concluded that different mechanisms of change may be at work in different trauma-related mental health responses in children [15]. A well conducted study, the major limitation of the Kilic study is the small sample size and the questionable generalizability of the findings to Western cultures.

The studies reviewed provide some evidence for impaired post-disaster family functioning [16] and for a relationship between impaired family functioning and post-disaster PTSD [14,15] and general anxiety [15] in children. However, methodological issues; the lack of well-validated measures of family functioning; lack of a direct measure of child post-traumatic stress symptoms and small sample size limit the usefulness of these findings and the interaction between family functioning and the child’s reaction to stress remains not well understood [20]. Our aim was to explore family functioning in a post-disaster environment. Specifically, we were interested in whether family functioning post-disaster would be impaired relative to a non-exposed community sample. In addition, we investigated associations between post-disaster family functioning and disaster-related constructs such as exposure, evacuation, and threat perception. Finally, analysing whether family dysfunction was differentially related to PTSD symptoms versus a more general (anxiety-depression) mental health response, as suggested by Kilic et al., [15].

## Methods

Far North Queensland, Australia is a large, sparsely populated (approximately 270,000 inhabitants in a 273,000 km^2^) region that supports agriculture, mining and tourism to the ‘outback’, coastal rainforest and the Great Barrier Reef. It is also a tropical cyclone prone area. When Cyclone Larry made landfall over the coast of North Queensland it was recorded as a Category 5 tropical Cyclone. Damage and destruction was severe; buildings in the region’s centre and surrounding townships suffered between 15 to 99% damage, and destroyed crops significantly impacted the income and livelihood of a majority of residents. The total insurance cost was estimated at $AUS 360 million. Considering the extensive damage to the built environment it is miraculous that no lives were lost.

### Procedure

Soon after the cyclone, Federal and State government funded NGOs (e.g. Australian Red Cross) provided the initial disaster response. By three months the response reverted to a Queensland government responsibility. At one month post-disaster Queensland Health, Education Queensland, Catholic Education and the research team agreed to screen and case identify students in the cyclone affected areas for persisting mental health problems. School-based screening has been successfully conducted in Australia following previous natural disasters [21,22]. Screening took place 3 months after the Cyclone. Following completion of a brief training module and standardisation process, school counsellors read out the screening questions to elementary school students. On completion of the questionnaires students were provided the opportunity to discuss their disaster experience within the class setting. All information collection followed signed parent consent, Catholic Education and Health departmental approval and University of Queensland HREC approval.

### Participants

Screening was offered to all school children in the designated disaster zone, total sample size 803, screening participation rates by schools varied from 35 to 64%. Parents of children attending catholic elementary schools were also asked to complete the family functioning measure. Approximately equal numbers of students participated from each grade (range 21.2% to 30.5%). Of 162 catholic primary school age children, parent-report FAD was available for 145 children (89.0%). There was no difference in the mean age (10.15 years, SD 1.24 versus 10.11, years SD 1.73, t = −1.03, p = 0.54), gender (47.1% female versus 46.9%, *χ*^2^(1) = 0.000, p = 0.99) or child PTSD symptoms (mean total PTSD RI score 21.90, SD 14.35 versus 20.00, SD 14.82, t = −0.515, p = 0.70) between those who did and did not complete the family measure. Similarly, there was no statistical difference of age, gender or PTSD symptoms between this sample and the remainder of the total screened sample.

There was no collection of family income or other SES-related data, primarily due to community sensitivity about research in the post-disaster environment. However, compared to Queensland as a whole, Indigenous Australians (7.7% of the Heath Service District population versus 2.3% in Queensland) and people living with relative socioeconomic disadvantage (36% versus 20%) were over-represented in this community [23].

### Measures

*Family Assessment Device (FAD)*[24]: the FAD is a self-report measure of family health and/or dysfunction. Family members rate on a four-point Likert-type scale how much they agree or disagree that their family environment matches scale statements. The full FAD consists of six subscales that correspond to the McMaster Family Assessment Model: problem solving, communication, roles, affective responsiveness, affective involvement, and behaviour control [25]. This research used parent-report of the seventh subscale, the General Functioning scale (FAD-GFS) which measures the families’ overall health and pathology [24]. Higher mean scores represent greater dysfunction; Miller and colleagues [26] established a cut-point of 2.00 for a diagnostic confidence rating of 0.83. The FAD-GFS has acceptable split-half reliability (Guttman’s split-half reliability r = .83) on a large sample of Ontario families [27], adequate concurrent validity [24,26] and discriminate validity [28]. Australian community normative data exist from a large (n = 2737) epidemiological study of mental health and well-being, conducted in collaboration with the Australian Bureau of Statistics and along with measures of mental health used the FAD-GFS to measure family functioning [29]. These data were used for comparison purposes in this study. The internal consistency in this sample was acceptable for research purposes (Cronbach alpha 0.88).

*The PTSD Reaction Index (PTSD-RI).* The Posttraumatic Stress Disorder-Reaction Index (PTSD-RI) [30,31] is an extensively used measure of traumatic stress in children and adolescents, especially following natural disasters [32]. The PTSD-RI consists of 20 items, worded to include a description of the name of the traumatic event. Respondents rate the symptoms experienced over the past month on a Likert scale from 0 “none of the time” to 4 “most of the time”. Item scores are summed to yield a total PTSD-RI score (range from 0 to 80). Cut-off scores allow severity level categorisation: total PTSD-RI score of 0 to 11 = ‘doubtful’, 12 to 24 = ‘mild’, 25–39 = ‘moderate’, 40–59 = ‘severe’ and greater than 60 = ‘very severe’. The PTSD-RI has acceptable psychometric properties for research purposes [13,22,30,33]. In the present sample the Cronbach’s alpha value was .90 and the Guttman Split-Half coefficient was .89.

*Other measures.* Non PTSD anxiety and depression psychopathology was measured with the parent-report 5 item emotional subscale of the Strengths and Difficulties Questionnaire (SDQ) [34,35]. Items are rated on a three-point response format (statements are 0 = Not True, 1 = Somewhat True or 2 = Certainly True) and include: (Over the 6 months my child…) “*Often complains of headaches, stomach-aches or sickness*”, “*Many worries, often seems worried*”, “*Is often unhappy, depressed or tearful*”, “*Is nervous or clingy in new situations, easily loses confidence”* and “*Has many fears, easily scared*”. The SDQ is considered a highly reliable and valid instrument for both screening and research purposes [34], see http://www.sdqinfo.com) of general internalizing symptomatology. A further parent-report SDQ item was included; whether their child had experienced pre-cyclone mental health problems (specifically, anxiety and depression).

The child screening questionnaire also included questions about the child’s disaster experience, perception of threat and the cyclone aftermath and recovery. Exposure questions included “Did you see flying debris?”; “Was your home damaged?”; “Did your home lose part of its roof?”; “… its whole roof?” and “Were any windows broken?” Children were also asked about their evacuation experience. Perception of threat was assessed by two questions: “Did you think you were going to die during the cyclone?” and “Did you think a family member might die during the cyclone?” Questions about the cyclone aftermath related to loss of possessions, needing temporary accommodation and whether any repairs on their home had been completed. Children indicated their endorsement of the questions using a “yes = 1” or “no = 0” response format.

## Results

Participating students were exposed to numerous frightening events: 53.9% of students reported some home damage, 26.7% reported losing part of their home’s roof, 17.9% were evacuated to a place deemed to be more safe, 11.8% had to live somewhere other than their home because of safety concerns or home damage. Many students (27.9%) thought they may have died during the cyclone. This is consistent with considerable child self-report of PTSD symptoms on the PTSD-RI: self-report of moderate symptoms 22.8%, severe or very severe symptoms 12.9%. The average PTSD-RI score was 21.70 (SD 14.37). The average SDQ-emotional subscale score was 1.49 (2.15), with 13.1% of children meeting case criteria for ‘abnormal’ on this measure.

Following the cyclone disaster, the mean parent-report FAD score was 1.63 (SD 0.44, min 1.00, max 2.91). Applying published cut-offs for family dysfunction, 41 parents (41/145, 28.3%) reported current family dysfunction. Parent-report of family functioning did not differ significantly with child gender. However, the mean age of children (10.70 years, SD 0.18) in dysfunctional families (Table 1) was significantly greater than the non-dysfunctional group (9.93 years, SD 0.11, t = 3.525, p < .000). Family dysfunction in this sample was significantly higher than the reported rate in a large community based Australian sample (28.3% versus 12.3%, *χ*^2^ = 30.79, p = 0.000).

**Table 1:** Bivariate associations between FAD case status and disaster-related events

	FAD	FAD	χ²	*p*
	‘case’	no-‘case’		
	n (%)	n (%)		
Age (years)	10.7 (0.2)	9.9 (0.0)	3.525^*^	0.00
Gender (female)	16 (39.0)	52 (50.0)	1.422	0.23
Home damage	23 (28.7)	57 (71.3)	0.000	0.98
Lose Part	13 (36.1)	23 (63.9)	1.349	0.24
Lose Whole	4 (40.0)	6 (60.0)	0.662	0.42
Evacuated	7 (25.9)	20 (74.1)	0.090	0.76
Saw flying debris	30 (27.8)	78 (72.2)	0.000	1.00
Threat perception	7 (17.1)	34 (82.9)	3.537	0.06
Temporary accommodation	7 (53.8)	6 (46.2)	3.919	0.05
Repairs complete	9 (68.9)	29 (64.2)	0.215	0.64
Possessions replaced	16 (40.7)	39 (48.7)	0.505	0.48
Previous difficulties**	17 (48.6)	18 (51.4)	10.399	0.00

* Students t test.

** Measured in months.

### Relationship of family dysfunction with disaster-related variables

There was no significant relationship between the parent’s or child’s report of disaster related exposures, such as home damage, home being destroyed or disaster-related evacuation, and family functioning at 3 months post disaster (Table 1). However, there was a trend for an association between the child’s perception of threat and less problematic family functioning (χ_1_^2^ = 3.537 P = 0.06, Fisher’s exact = 0.07, see Table 1). At multivariate analysis there was an independent, significant relationship between the child’s threat perception and abnormal family function (OR_adj_ 0.26, 0.09-0.79). That is there was more family dysfunction in the group who did not experience threat perception during the cyclone.

Questions that related to post-disaster difficulties did not demonstrate a significant association with family functioning. For example, at the time of screening, 65 parents stated their home had been damaged and repairs had not been completed. Family dysfunction (total score or dysfunction ‘case’ membership) was not greater in this group (t = 0.150, p = 0.87; χ_1_^2^ = 0.00, p = .95). Similarly, children who cited they had lost possessions were not more likely to be in the abnormal family functioning group (t = 1.453, p = 0.14; χ_1_^2^ = .75, p = 0.39).

### Post-disaster family functioning and relationship with PTSD

The total PTSD symptom score in children from families who did not meet case criteria for family dysfunction was not significantly different to families that met criteria (19.27, SD 1.95 versus 22.94, SD 1.46, t = 1.392, p = 0.16; see Table 2). There was also no difference when comparing FAD case status and whether the child was in the severe-very severe PTSD symptom group or not (*χ*^2^ = 0.371, p = 0.54). To account for a relationship emerging at more extreme levels of family dysfunction the data was re-analysed a different FAD cut-off points. In families whose FAD total score was 2 standard deviations (towards dysfunction) from the mean there was no significant difference in child PTSD scores (PTSD-RI 22.15, SD 14.5 versus 20.42, SD 13.5, t = 0.508, p = 0.61). Similarly, there was no difference when 3 standard deviations from the mean (PTSD-RI 22.10, SD 14.5 versus 19.75, SD 13.6, t = 0.541, p = 0.59). The lack of relationship remained following other techniques to account for non-standard distributions, such as log and quadratic transformations.

**Table 2:** **Bivariate relationships between family functioning ‘case’ status and post-disaster child PTSD1and emotional symptoms2**

		PTSD-RI^1^		
		score (SD)	t	p
FAD	case	19.27 (1.95)	1.392	0.16
	non-case	22.94 (1.46		
		**PTSD-‘case’**		
		**n (%)**	**χ**^**2**^	**p**
FAD	case	4 (22.2)	0.371	0.54
	non-case	14 (17.8)		
		**SDQ-Emot**		
		**Score (SD)**	**t**	**p**
FAD	case	2.66 (0.40)	3.738	0.00
	non-case	1.15 (0.19)		
		**SDQ-‘case’**		
		**n (%)**	**χ**^**2**^	**p**
FAD	case	10 (52.6)	6.95	0.01
	non-case	9 (47.4)		

^1^ PTSD-RI total score.

^2^ Strengths and Difficulties questionnaire, Emotional subscale.

### Relationship of family dysfunction with internalizing child symptoms

The mean score of the parent-report emotional subscale of the SDQ-Em was significantly higher in families reporting dysfunction than it was for the non-dysfunctional group (Table 2). There was no significant relationship with parent-report on the SDQ-Em and pre-existing mental health problems (*χ*^2^ = 1.39, df 141, p = 0.24). Family functioning was strongly associated with parent report of past child emotional difficulties and with longer duration of these difficulties (Table 1).

Independent statistical contributions to adverse post-disaster family functioning was assessed by logistic regression and included: (model 1) previous mental health difficulties and SDQ emotional symptoms; factors related to the disasters (model 2) such as home damage, child PTSD symptom category and the child’s threat perception and a final model (model 3) of all factors significant at the p > 0.1 level (Table 3). All models were adjusted for child age and gender. In the final model, increasing child age, greater child emotional difficulties on the SDQ-Em scale and no child perception of threat during the cyclone remained independent significant predictors of abnormal post-disaster family functioning.

**Table 3:** Logistic regression: Multivariate relationships with Family Functioning

	Model 1	Model 2	Model 3
	OR (95% CI)	OR (95% CI)	OR (95% CI)
Gender	0.43 (0.14-1.31)	0.65 (0.30-1.43)	0.70 (0.29-1.66)
Age	1.71^*^ (1.05-2.80)	1.76^**^ (1.24-2.50)	1.78^**^ (1.24-2.57)
Prev. difficulties	6.42 (0.45-2.02)		
Difficult >12 mths	0.76 (0.30-1.99)		
SDQ-Em score	1.40 (0.98-2.00)		1.46^***^ (1.20-1.78)
Temp. accomodatn.		1.42 (0.32-6.33)	
PTSD category		1.37 (0.36-5.18)	
Home damage		0.96 (0.44-2.09)	
Threat to self		0.40 (0.15-1.09)	0.26^*^ (0.09-0.79)

Significance: odds ratios in bold, 95% CI do not include 1.00 and ^***^ p < .001, ^**^ p < .01, ^*^ p < .05.

Model 1: Mental health and social connectedness.

Model 2: PTSD, threat and exposure factors.

Model 3: (final) all model 1 and 2 variables with p < 0.10.

## Discussion

The first finding of note was that using established community cut offs for the FAD–GFS, as hypothesized, there was a significant elevation in the number of parents reporting family dysfunction in the post disaster period. Considering the relationship between post-disaster family functioning and disaster-related variables, in contrast to McFarlane’s [16] findings, exposure to disaster related events (evacuation experience, home damage, repairs not completed 3 months post disaster) did not differentiate dysfunctional from non-dysfunctional families. In understanding our different finding we used a validated, psychometrically sound measure of family functioning and the present study was conducted at 3 months rather than 8 months post-disaster. It is possible that in the aftermath of a disaster, it is only once the most pressing practical tasks have been completed that the differential impact of disaster exposure is experienced. The degree of disaster exposure was not the reason why parents in this study reported higher levels of family dysfunction compared with a community sample. The issue of timing; which variables are associated with different outcomes at different time points after a disaster is an interesting question for future research. Children who did not perceive themselves to be under threat of death during the cyclone were more likely to belong to a family rated by parents as dysfunctional. This is an intriguing, and somewhat counter-intuitive finding and warrants further study.

The third aim of the current study was to investigate whether family dysfunction was related to PTSD or general, internalizing mental health symptoms. In contrast to McFarlane [16] and Green et al., [14], but consistent with Kilic et al., [15], we found no independent significant relationship between post-disaster family dysfunction and child PTSD. However, children whose parents rated them as higher on internalizing symptoms post-disaster were more likely to come from families rated by parents as dysfunctional. This finding is partially consistent with Kilic and colleague’s results, where a relationship between family dysfunction and anxiety, but not depression, was reported.

In attempting to understand these findings, we also considered the possibility that elevated internalizing symptomatology may have preceded the disaster. However, this was not the case – children whose parents rated them high on internalizing symptoms post-disaster were no more likely than children rated low on these symptoms to have had any pre-existing mental health problems. Three months after the cyclone, parents were able to make an association between elevated family dysfunction and elevated child internalizing symptomatology while not making the association between family dysfunction and PTSD symptomatology. Why would children’s post-disaster internalizing symptoms but not their PTSD symptoms be related to family dysfunction? Extending Hobfoll’s theory to consider post-disaster family functioning, families clearly experience both actual (e.g. financial) and threat of resource loss. Loss may be of competence, for example a parent’s emotional accessibility due to their own post-disaster mental health symptoms. This is consistent with our report of elevated family dysfunction. Children may respond to this dysfunction with greater internalising symptoms. Disaster-related losses may, independently, lead to other presentations e.g. PTSD. This is consistent with Kilic and colleagues; based on their small sample of Turkish families they suggested that different mechanisms of action may underlie different post-disaster mental health responses in children. Another possibility lies in our understanding and conceptualization of PTSD. Recent research has demonstrated that dysphoria or general distress underlies internalizing disorders in adults – including PTSD [36,37]. In a recent factor analytic investigation of PTSD symptoms in an epidemiologically based trauma-exposed sample from Australia [38], empirical support was found for an inter-correlated four-factor model that includes dysphoria as well as the existing 3 symptom clusters. It may be that it was children’s distress rather than the more traditional diagnostic features of PTSD that had ‘registered’ with parents and been associated with perceived family dysfunction and applying a four-factor model of PTSD may have found an association with family dysfunction.

There are several limitations of this study. Little is known about families who did not consent to the screening process. There may be a sample selection bias with this group demonstrating a more significant traumatic stress-family dysfunction association. In the methods section limited collection of SES data was noted, due to ‘research sensitivity in the post-disaster environment’. Our approach is to integrate research questions with screening, case identification and offering therapy if appropriate. This process of local involvement of parents and school communities in service decisions (i.e. screening) is well accepted by parents and this collaboration facilitates research, especially research questions related to service provision. Whilst it was fortunate this disaster resulted in no loss of life, our results therefore cannot be immediately generalised to events where bereavement is widespread. Another limitation is the variation in reporting source. Thus, child PTSD was assessed via child-report, whereas family functioning and child internalizing symptomatology were assessed via parent-report. The study would have been improved by the use of more targeted measures of child anxiety and depression. A broader focus and measuring other child outcomes, most notably disruptive behaviour and substance use may be informative. It is possible that children with disruptive behaviour disorders, often from families with elevated dysfunction, may be more resilient to post-disaster PTSD as a consequence of greater lifetime exposure to conflict and frightening events. Future research could also assay the child’s opinion of pre and post-disaster family functioning and compare with parent perception. Finally, we emphasise our findings are statistical associations; a longitudinal model is needed to better describe causal relationships.

## Conclusion

Our research provides some preliminary evidence that, in the short-term aftermath of a natural disaster, there may be a higher frequency of discordant family functioning. The results also suggest that different mechanisms of action may underlie different post-disaster mental health responses in children. Broadening data collection to include post-disaster changes in parenting style, child disruptive behaviour and in adolescents substance abuse, is indicated.

## Competing interests

The authors declare they have no competing interests.

## Authors’ contributions

Both authors contributed to study design and methodology. BMcD was primarily responsible for statistical analysis. Both authors read and approved of the final manuscript.

## Pre-publication history

The pre-publication history for this paper can be accessed here:

http://www.biomedcentral.com/1471-244X/12/55/prepub
